# Fulminant myocarditis: a comprehensive review from etiology to treatments and outcomes

**DOI:** 10.1038/s41392-020-00360-y

**Published:** 2020-12-11

**Authors:** Weijian Hang, Chen Chen, John M. Seubert, Dao Wen Wang

**Affiliations:** 1 Division of Cardiology, Department of Internal Medicine, and Hubei Key Laboratory of Genetics and Molecular Mechanisms of Cardiological Disorders, Tongji Hospital, Tongji Medical College, Huazhong University of Science and Technology, Wuhan, 430030 China; 2 Faculty of Pharmacy and Pharmaceutical Sciences University of Alberta, Edmonton, Alberta T6G 2E1 Canada

**Keywords:** Cardiology, Inflammation

## Abstract

Fulminant myocarditis (FM) is characterized by a rapid progressive decline in cardiac function and a high mortality rate. Since the first report of FM patients in the 1980s, several clinical trials and research studies have been published increasing our knowledge regarding FM. Currently, the diagnosis of FM depends on various techniques including electrocardiography, echocardiography, endomyocardial biopsy, and cardiac magnetic resonance. The development of mechanical circulation support (MCS) devices and progress in our understanding of the pathophysiological mechanisms underlying FM, treatment regimens have evolved from simple symptomatic treatment to a life support-based comprehensive treatment approach. The core mechanism underlying the development of FM is the occurrence of an inflammatory cytokine storm. This review provides a comprehensive account of the current understanding of FM pathophysiology and knowledge regarding its etiology, pathophysiology, treatments, and outcomes.

## Graphical abstract

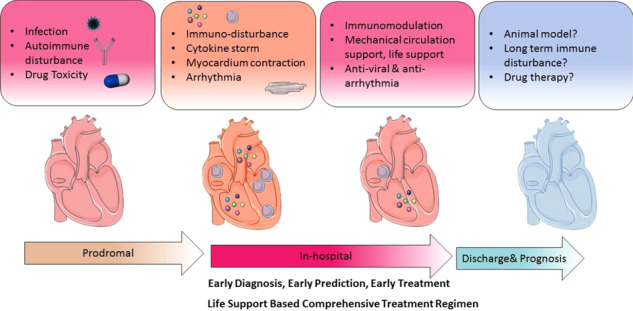

## Introduction

Fulminant myocarditis (FM) is an uncommon but severe cardiac inflammatory disease that can be fatal.^1,2^ The progressive nature of the disease is characterized by a severe and sudden onset, which is marked by a rapid deterioration within 2 weeks that can occur within 2 or 3 days.^3^ The rapid decline makes it difficult to obtain a clear and early diagnosis, which often leads to misdiagnose or delay in diagnosis prior to a patient’s death. Once FM is suspected or diagnosed, use of modern life support devices is highly recommended to prevent loss of life.^4,5^ Recent improvements in FM treatments have resulted in decreased mortality rates from over 50%^6^ to <5%^7,8^ in distinct clinical centers, however, detailed knowledge of the pathogenesis remains limited. The aim of this review is to present an integrated account of the latest knowledge about FM, to improve disease understanding and to provide advice to physicians regarding its treatment and possible outcomes.

## Clinical manifestations and evaluation of FM

FM is a clinical diagnosis where prodromal symptoms, such as fatigue, cough, dyspnea, and chest pain often show no distinguishable difference from the common cold. An important feature of FM is its rapid clinical progress, which can quickly lead to hemodynamic dysfunction and circulation instability. This is presented as a sharp drop of blood pressure that cannot be maintained properly by vasoactive drugs and requires mechanical circulation support (MCS) devices. It is common for multiple organ failure to be observed in FM patients. As several reports about FM indicate, deterioration and collapse of the circulatory system can occur as rapidly as 2 days to 2 weeks from the onset of precursor symptoms, highlighting the importance of early and differential diagnosis of FM to ensure early treatment application.

When a suspected patient is admitted, routine tests including physical examinations, blood chemistry, 12-lead electrocardiogram (ECG), and emergency echocardiography should be conducted.^4^ Physical examinations may reveal signs of heart failure, including decreased blood pressure, accelerated heartbeat, and markedly decreased heart sounds, usually with gallop rhythm, with or without tachypnea. Routine blood tests may reveal elevated neutrophils or lymphocytes, indicating the presence of infection. Viral serological assays should be considered but importantly negative results do not necessarily rule out possible viral infection.^9^ ECG tracings demonstrating arrhythmias or tachycardia with sinus rhythm can demonstrate left bundle branch block (LBBB), reduced QRS wave amplitude, ventricular premature beat, and ventricular tachycardia suggestive of severe progression.^10^ Considering the results of echocardiography can vary according to the state of the FM patient’s heart function, repeated and close monitoring is recommended. Markers of myocardium injury, such as cardiac enzymes and troponin I or T, as well as NT-ProBNP provide insight into damage and cardiac dysfunction but both acute coronary disease and FM can have elevated biomarkers. As such, in order to differentiate FM from acute coronary disease, emergency angiography should be considered.

When a patient presents symptoms of hemodynamic compromise, it is very important to monitor the hemodynamic status with an electric blood pressure recorder or in-vessel blood pressure monitor and echocardiography. Compared to CMR, echocardiography is an easier approach in common hospital settings limiting operational complexity. In addition, the development of the global longitudinal strain (GLS) techniques, allows better assessment of heart function and provides an index for prognosis as observed in immune checkpoint inhibitor-related myocarditis.^11^ It is worthy to investigate the value of GLS in FM in the future, but it is not easy to perform in the acute phase because of the critical conditions of patients.

## Etiology

While the exact etiology of FM remains largely unknown, our current understanding indicates three main factors contribute to its development. The first factor suggests an infection caused by various pathogens, especially viruses,^2,12^ which manifest in clinical features observed in FM. In fact, certain types of viral nucleic acids can be detected directly in endomyocardial biopsy (EMB) samples and serum by real-time polymerase chain reaction (PCR) or in situ hybridization (ISH),^9,13^ such as parvovirus B19 (PVB19), coxsackievirus B3 (CVB3), and cytomegalovirus (CMV).^2^ Although uncommon, some viruses that usually infect non-cardiac organs, like human immunodeficiency virus (HIV),^14^ can also induce FM. Importantly, due to the limited sensitivity of detection assays, it is possible to get false-negative results of a viral infection. A multicenter study revealed that only 38% of myocarditis patients can find viral genome in their EMB samples.^15^ PCR or PCR-based detection techniques show the best performance, reaching sensitivity levels in the range of 50–90%,^9,13,16^ and an ability to detect multiple viruses in clinical samples.^17^ In contrast, serological tests and ISH of EMB samples show poorer sensitivity.^9^ A study reported a poor correlation between PCR and serological results, with only 4% of serological evidence of viral infection being determined by EMB.^9^ However, PCR-based detection techniques are limited in scope to predicted or suspected viruses due to the need for specific primers for amplification. Recent advances with next-generation sequencing (NGS) has provided clinicians with an ability to obtain unbiased results of possible pathogens of infectious myocarditis.^18,19^ Interestingly, several uncommon viruses including Epstein Barr virus (41%), human pegivirus (4%), human endogenous retrovirus K (100%), and anellovirus (56%) were found indicating the complexity of the viral constitution in FM. Apart from viruses, bacteria including *Chlamydia pneumoniae*, *Mycobacterium tuberculosis*,^12^ *Neisseria meningitidis*^20^ or protozoan such as *Plasmodium falciparum*, *Toxoplasma gondii*^12^ or *Giardia lamblia*^21^ have all been reported to trigger FM. Hence, special attention should be paid to infected patients with rapid deterioration of cardiac function.

The second factor contributing to FM development is autoimmune disease. The systemic lupus erythematosus (SLE),^22^ rheumatism,^23^ scleroderma^24^, and Sjogren’s syndrome^25^ have all been reported to induce FM. By expressing PD-L1 on myocardium, the heart is considered to be well-protected from cardiac-specific T cells, which mostly recognize the α-myosin heavy chain peptide.^26^ However, a disturbance of the balance of immune homeostasis by autoimmune diseases may enable self-antigens to be exposed to the immune system and mistakenly activate myocardium cytotoxic T cells. This may partially explain the effects of glucocorticoids in treating autoimmune disease-induced FM.^27^ Since the key pathological process of autoimmune disease-induced FM is self-antigen such as alpha or beta-MHC exposure to the immune system, it is convincing that disturbances of the immune system caused by autoimmune diseases lead to the formation of auto-cytotoxic immune cells, CD3^+^/CD8^+^ T cell predominantly, as well as macrophages, targeting the myocardium, eventually leading to FM.^28^

The third factor contributing to FM development is drug toxicity. Many drugs, especially chemotherapy drugs and certain natural derivatives,^29^ are toxic to cardiomyocytes,^30,31^ which is exemplified by increased incidences in check-point inhibitor-related FM.^32^ Although check-point inhibitors have brought revolutionary advances to the treatment of late-stage malignant cancers they induce the formation of auto-cytotoxic immune cells. These cells subsequently attack the myocardium, resulting in the accumulation of CD3^+^CD8^+^ T cells, macrophages, and neutrophils in the heart.^33,34^ The prevalence rate of cardiac side effects of checkpoint inhibitors is common, which can reach 25% or more.^35^ Although the prevalence rate of FM from checkpoint inhibitors is <1% and is much lower than other targets, its fatality rate is as high as 40~70%.^35–37^ Therefore, it is important to pay attention to patients receiving chemotherapy who display cardiac function deterioration, as this may be a sign of FM.

To date, there are three major classes of etiological factors in FM known (Table 1). However, the exact mechanism(s) behind each etiological factor remains largely unknown. Importantly, these factors are not completely distinct but have overlapping signaling pathways and cellular responses triggering their effects. It may be hypothesized that the mechanisms underlying FM involve common immune system pathways, for example, check-point inhibitor-induced FM and hypersensitivity induced FM, are related to auto-immune disruption and myocardium cytotoxicity T cell activation.^38,39^

**Table 1:** Major etiological factors and agents of fulminant myocarditis

Factors	Underlying agents
Infection	• Virus (Most common): Coxsackievirus B3 (CVB3), Parvovirus B19 (PVB13), Adenoviruses, Herpesviruses, HIV, Influenza A, etc.; • Bacteria: *Chlamydia pneumoniae, Mycobacterium tuberculosis, Neisseria meningitidis*, etc.; • Spirochete: *Borrelia burgdorferi*, etc.; • Parasite: *Plasmodium falciparum*, *Toxoplasma gondii*, *Giardia lamblia*, etc.
Autoimmune disturbance	• Autoimmune disease: Systemic lupus erythematosus (SLE), Rheumatism, Scleroderma, Sjogren’s syndrome, Inflammatory bowel disease, Churg-Strauss syndrome, etc.; • Sarcoidosis, rare but need special attention;
Drug toxicity	• Chemotherapeutic drugs: Anthracycline etc.; • Drug hypersensitivity: Cephalosporin, Digoxin, Clozapine, etc.; • Allergy: nickel. • Plant derivatives: *Aconite*, *Garcinia Cambogia* extract; • Checkpoint inhibitor.

## Pathological classifications of FM

**Fig. 1:**
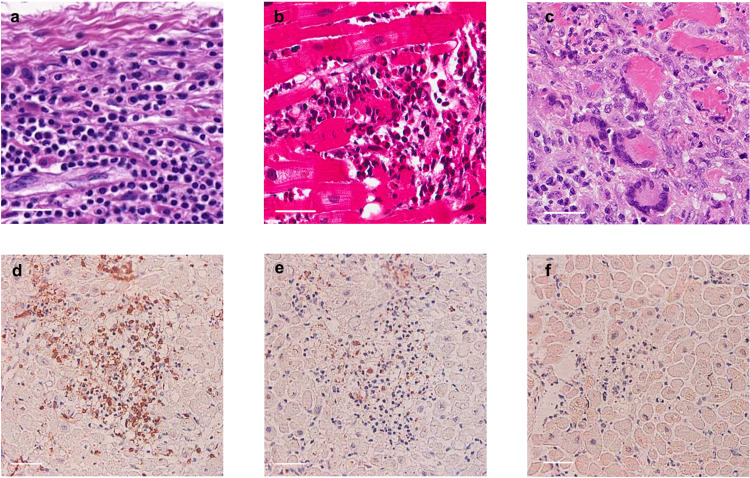
Different pathological phenotype of FM. **a**–**c** representative HE staining of EMB samples of FM patients showed lymphocyte FM (**a**), eosinophilic FM (**b**), and giant cell FM (**c**). **d**–**f** IHC staining showed massive T lymphocyte (CD45RO) infiltrated into myocardium (**d**). Macrophage (CD68) can also be observed (**e**). Few B lymphocyte (CD20) can be seen in EMB samples (**b**). Figure 1d–f is from ref. ^130^ with the permission of *Circulation Journal*; Fig. 1b is from ref. ^153^ with the permission of *ESC Heart Failure*; Fig. 1c is from ref. ^49^ with the permission of *BMJ Case Report*

Although FM is thought to be a clinical diagnosis, it can also be based on histopathological findings. The improvement in EMB techniques has permitted clinicians the ability to make safe and precise pathological diagnosis of FM.^1^ The pathological classifications of FM are still under the framework of the Dallas Criteria, according to which FM is classified as lymphocyte myocarditis, eosinophilic myocarditis and giant cell myocarditis (Fig. 1). Although rare, cardiac sarcoidosis is a special pathological type of FM, whose incidence is tightly related to genetic background.^40^

It is recommended to obtain a minimum of four or five samples to limit sampling error^41,42^ when performing histopathological examinations. Standard hematoxylin eosin (HE) staining can directly reveal the pathological phenotype but immunohistochemistry (IHC) can be applied for further identification.^5^ IHC analysis will distinguish between morphological changes that are due to the sampling process and the actual morphological shapes of infiltrated immune cells.^43^ Moreover, IHC using cell type-specific markers can make accurate classifications and help distinguish the sub-types of infiltrated cells, such as the different T lymphocyte subtypes (CD4^+^, CD8^+^), macrophage (CD68^+^), and B lymphocyte (CD20^+^). Transmission electron microscope (TEM) can provide myocardium ultrastructure information and possibly identify viral particles,^44^ but technical limitations slow result acquisition and is not used as a routine test.

The importance of the pathological classification of FM goes beyond simply providing a pathological diagnosis. A pathological classification will provide information about disease prognosis^45^ as well about its etiology and treatment guidance. For example, data demonstrating lymphocyte infiltration often implies infection, usually by a virus.^10^ While evidence of eosinophil infiltration indicates an allergy, a parasite infection^46^ or a drug reaction with eosinophilia and systemic symptoms syndrome (DRESS syndrome).^47^ And the formation of giant cells is often related to autoimmune disease or sarcoidosis.^48,49^ Both eosinophils and massive lymphocytes can be observed in parasite-induced FM.^50^

Important information garnered from pathological assessments can provide guidance toward therapeutic decisions. For example, immediate immunosuppressive treatment with glucocorticoids and subsequent tapering to low maintenance dose may be initiated based on evidence of giant cell FM, cardiac sarcoidosis, and eosinophil myocarditis resulting in better outcomes.^51,52^ However, the application of glucocorticoids in the treatment of lymphocyte FM remains debatable, notably, disease caused by viral infections. Evidence against the use of glucocorticoids in the treatment of lymphocyte FM reflects concern the virus infection may worsen and spread due to glucocorticoid-induced immunosuppression.^5^ While others have shown glucocorticoid use could reduce virus titer by stimulating interferon secretion.^8,53^ Thus, highlighting the importance in considering the complexity of FM pathogenesis and its exact etiology as different clinical approaches must be carefully determined based on a comprehensive analysis of clinical history, lab auxiliary test results and EMB pathological diagnosis.

## Pathophysiological mechanisms underlying FM

The lack of comprehensive and systemic knowledge regarding the pathophysiological mechanisms underlying FM has limited therapeutic treatment regimens hindering effective interventions at different disease stages. Although significant progress has been made in our understanding persistent efforts are still required to elucidate the complete pathophysiological mechanisms involved.

### Cytokine storm in FM

While the complete etiology of FM remains unknown, a dysregulated immune response has a critical role in the development of FM. Adverse effects caused by infectious pathogens can overstimulate the immune response contributing to the rapid disease progression. Evidence from EMB samples demonstrate numerous infiltrating immune cells in the necrotic myocardium.^54^ According to most reports, the majority of infiltrated cells are T lymphocytes, macrophages, and rarely B lymphocytes. Detection of CD3^+^CD4^+^ Treg lymphocytes or CD3^+^CD8^+^ cytotoxic T lymphocytes^55^ is commonly observed in EMB samples, which is consistent with the IHC results obtained from FM patients at our center, demonstrating massive lymphocyte infiltration (Fig. 1). Other immune cells reported to infiltrate into myocardium obtained from FM patients include Treg and Th17 cells.^56,57^ In addition, lymphocyte myocarditis, eosinophilic myocarditis, and giant cell myocarditis are the result of significant immune disturbances. Degranulated eosinophils can be detected in the myocardium, suggesting key factors may be secreted affecting the local immune response,^27,50^ however, the exact components involved remain unknown. Phenotypic differences in human leukocyte antigen (HLA) are reported to produce different immune responses triggered by the same stimulus, which suggests genetic variants might be related to different sensitivities to FM.^40^

**Fig. 2:**
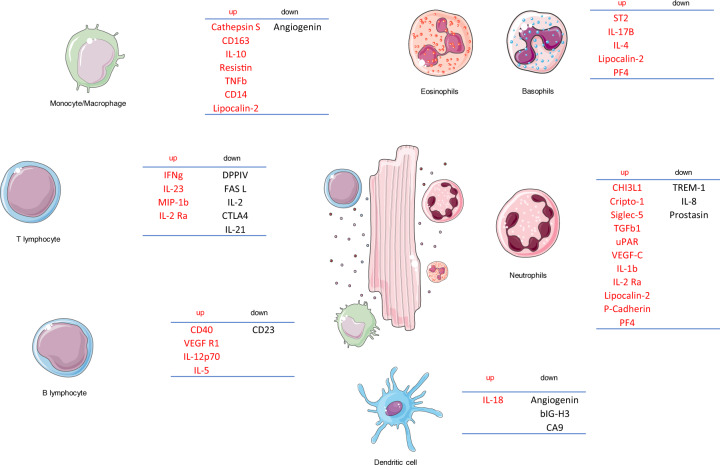
Cytokine storm of FM patients. Changed cytokines and their cellular origination. After scanning for changed cytokines, their immune cellular origin was searched using the Human Protein Atlas database (http://www.proteinatlas.org). Neutrophils and monocytes accounted for the majority of elevated cytokines and the majority of known immune cells participated in the dysregulation of cytokine profile

In our center, we have analyzed the serum of 4 FM patients to assess their cytokine profile. The data demonstrated marked alterations in the cytokine profile and concentrations in FM patients compared to healthy controls (Fig. 2). The majority of cytokines were upregulated but several cytokines were downregulated. Secretion of dysregulated cytokines occurred from numerous different immune cells (e.g., neutrophils, monocytes and lymphocytes) indicating involvement and disturbance of the whole immune system in the early phase of FM. In this context, we refer to the term “cytokine storm” to describe the disturbed immune homeostasis caused by FM. Importantly, these data highlight the fact a cytokine storm has already started in most FM patients at the time of admission due to a prolonged referral process. This early response contributes to a quick deterioration observed in a patient’s health immediately after admission. Interestingly, our data from FM patients demonstrated specific cytokines such as IL-1b, IL-4, IL-17B, IL-23, IL-10, IL-18, ST2, and IFNγ are significantly upregulated but will subsequently decrease to normal levels after proper treatments, which suggests they may be potential biomarkers (unpublished data). Therefore, clinically, investigations into the cytokine profile of FM patients can broaden our knowledge about the underlying pathophysiological process of FM and guide therapeutic decisions.^58^

**Fig. 3:**
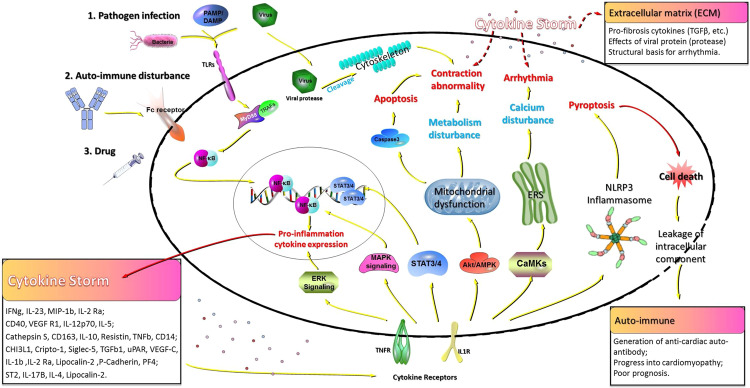
Illustration of signaling transduction in the development of a cytokine storm in FM and potential cardiac effects. Various etiologies trigger inflammatory signaling that result in transcription and translation of pro-inflammatory cytokines. Secreted cytokines activate numerous pathways via specific receptors leading to different cellular responses. Pathogens (e.g., viruses) can directly target cells resulting in marked damage worsening the prognosis of FM patients

Activation of cytokine ‘storm’ can be triggered by various etiologies including pathogens such as viruses,^59^ bacteria,^60^ spirochete^61^, and fungi^62^ (Table 1), which can initiate effects through receptors, like toll-like receptors (TLR). For example, leakage of intracellular components from damaged cardiomyocytes trigger an innate immune responses by activating TLR4.^63^ Once activated, downstream signaling cascades transfer information about the extracellular pathogen into intracellular transcription factors such as NF-κB^64^ and STAT3^65^ to elicit a cellular response. During the recent COVID-19 pandemic, several groups have reported SARS-CoV-2 induced FM^66^ and a subsequent cytokine storm.^67^ The upregulated cytokines activate receptor-mediated signaling^68^ pathways that increase ERK^69^ and MAPK^70^ activity and key transcription factors like NF-κB and STAT3, which result in further cytokine expression leading to a continual cycle of cytokine production. The impact of cytokine overproduction has direct effects on myocardium contraction and electrical transduction, which will be discussed below. Thus, the cytokine storm plays a central role in the pathophysiology of FM (Fig. 3), detailed reviews of the effect cytokines have on the heart can be found in the subsequent discussion and elsewhere.^65,71^

### Abnormal myocardial contraction

**Fig. 4:**
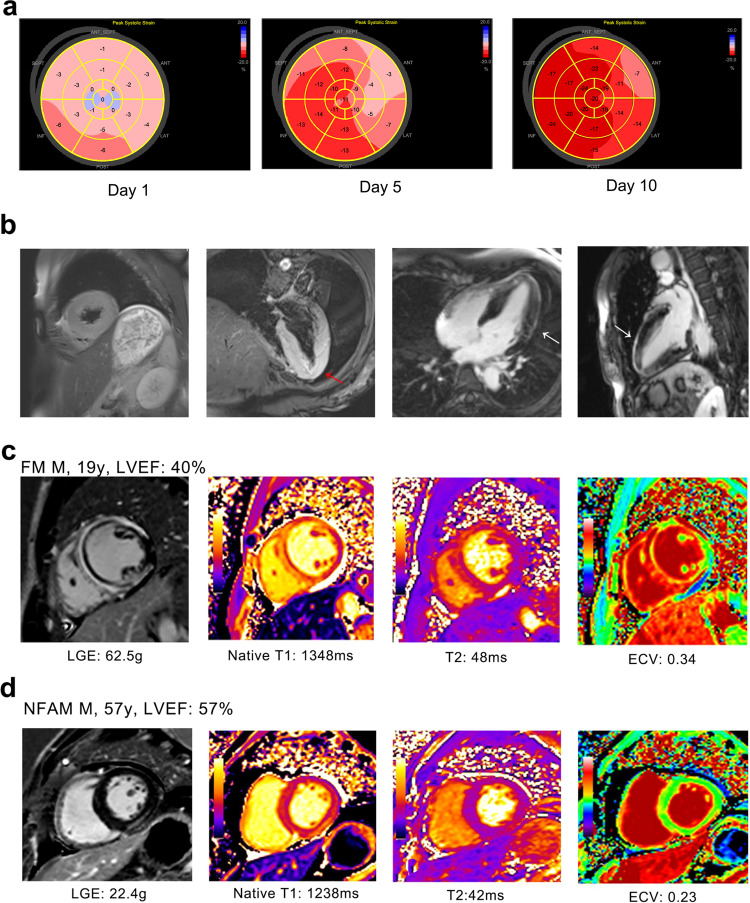
Representative echocardiography and CMR assessment images of clinical FM patients. **a** regional strain distribution of FM patients on the day of admission, the fifth day and tenth day of hospitalization. Note the regional strain distribution improved following appropriate treatment under the guidance of life support-based comprehensive treatment regimen. **b** Representative CMR image of FM patients. T2WI showed left ventricular hypertrophy and massive left ventricular wall edema (red arrow) in a 37-year-old FM female patient. The middle section of apex and left ventricular wall showed late gadolinium enhancement (LGE) signal (white arrow), which indicates massive myocardial injuries. **c**, **d** Representative CMR image of a FM patient (**c**) and a non-fulminant myocarditis patient (**d**). Note the increased diffuse LGE pattern of FM compared to non-FM patient. Longer native T1 and T2 and higher extracellular volume fraction (ECV) were observed in FM patients. Figures 4c and d is from ref. ^104^ with the permission of the *Journal of Magnetic Resonance Imaging*

The primary function of the heart is to pump blood throughout the body within the circulatory system. FM patients are characterized by a compromised circulation, which usually results in refractory cardiac shock requiring mechanical circulatory support (MCS). Detection of myocardial contraction abnormalities observed during the development of FM is routinely performed by echocardiography (Fig. 4)^72^ or cardiac magnetic resonance (CMR) (Fig. 4).^73^ Hallmark injuries like massive ventricular wall hypokinesia and edema are believed to be induced by a cardiac inflammatory response and global cytokine storm.

Cytokine storms disturb immune homeostasis and will directly influence the myocardium. It was reported pro-inflammatory cytokines, like IL-1 and tumor necrosis factor-alpha (TNF-α), have a negative ionotropic effect and directly decrease myocardial contraction strength and velocity.^74^ These effects provide insight into two common manifestations observed in FM. First, a rapid reduction in cardiac pump function and cardiogenic shock, represented by significant ventricular hypokinesia. And second, deteriorated heart function, as determined by decreased ejection fraction (EF%) or GLS. Depending upon the timing and therapeutic approach, dysfunctional hearts can return to normal or just slightly below the normal heart function range after appropriate treatments.^27,72,75^ Since myocardial cells are terminally differentiated and cannot be replaced once damaged, the normalization of heart function is probably due to a contraction improvement of each myocardial cell. Interestingly, a cytokine storm might cause myocardial stunning and decreased cardiac function without marked cell death, which could be reversed in a relatively short time once the stressor is removed.^75^

Regular myocardium contraction is dependent upon mitochondrial function to produce energy. However, adverse consequences of a severe cytokine storm produced during FM can directly inhibit mitochondrial function^76^ and reshape the metabolic status of the heart.^77,78^ An et al.^79^ used a LPS-stimulated ex vivo myocarditis model to demonstrate the secretion of TNFα and phosphorylation of NF-κB correlated with the depressed left ventricular contractile ability and H_2_O_2_ production in cardiac mitochondria. Accumulation of damaged or dysfunctional mitochondria will markedly disrupt energy supply, increase ROS production and activate innate immune responses, which ultimately lead to loss of cardiomyocytes and decreased heart function.^78,80^

Pathogens can directly target the myocardium inducing damage by releasing enzymes such as proteases or collagenases that target sarcomeric proteins^81^ and the extracellular matrix (ECM).^82^ Degradation of sarcomeric proteins like troponin and dystrophin will directly damage the normal structure of the sarcomere and fracture the myocardial filament. Breakdown of the myofilament results in an inability to transduce the contraction strength of the heart diminishing its ability to efficiently pump enough blood into the circulation. While the degradation of collagen fibers found in the ECM will stiffen the heart and reduce its elasticity further worsening the ability to contract.^83^

At the cellular level, pathogens have been demonstrated to activate different cell death pathways including apoptosis,^84^ necrosis, pyroptosis,^85,86^ and necroptosis^87^ resulting in decreased viability. Whether a cause or effect, activation of different cell death pathways are proposed to be involved in the pathogenesis of FM, even myocardium necrosis has been observed in EMB samples.^88^ Importantly, loss of terminally differentiated cardiomyocytes will significantly reduce the heart’s contraction ability. In addition, the increased cell death in the myocardium will further trigger inflammatory responses and exposure of self-antigens worsening the condition. This may subsequently lead to auto-immune cardiomyopathy,^89^ which is recognized as a poor prognosis factor in FM.^90^

### Abnormality of the cardiac electrical transduction system and arrhythmia

Maintaining coordination of electric transduction and synchronicity of the heart beat is of great importance for hemodynamic stability. However, arrhythmias are very common in FM and indicate a bad prognosis.^91^ Excitation-contraction coupling can transform the electric signal to a physical contraction by manipulating intercellular calcium signaling. Any disturbance in this process may result in development of an arrhythmia.

Unfortunately, the cytokine storm in FM interferes with normal calcium signaling. TNF-α and other pro-inflammatory cytokines can activate calcium/calmodulin-dependent protein kinase alpha (CaMKα) and the following NF-κB pro-inflammation pathway.^77,92^ High concentrations of TNF-α decrease calcium transients and attenuate cardiomyocyte contraction.^93^ IL-1 can activate ryanodine receptor (RyR) and release calcium from the sarcoplasmic reticulum, which causes intracellular calcium overload and deteriorates the excitation-contraction coupling process.^94^ Together, these changes will contribute to the development of arrhythmias.

Dysregulation of the calcium signaling pathways in cardiomyocytes and the infiltration of immune cells, like lymphocytes, during sarcoidosis is an important factor in the development of arrhythmias. Evidence from endomyocardial electroanatomic mapping studies indicate focal sites with massive lymphocyte infiltration have lower potential. Hence, it may be easier for the physician to obtain positive EMB samples under the guidance of endomyocardial electroanatomic mapping.^95^ Although rare, refractory arrhythmias are a characteristic of cardiac sarcoidosis, which contributes to its poor prognosis.^40^ Sarcoidosis may be complicated by cardiac fibrosis, which provides a structural basis for reentry of the electrical signal.^96^ Another common feature of FM is early repolarization (ERP), which may be a predictor of ventricular tachyarrhythmias. However, a recent study showed ERP was not associated with a worse prognosis and could not predict the development of lethal ventricular tachyarrhythmias.^97^

Indeed, it is important to precisely assess the electrical properties of the myocardium, especially where and when arrhythmia might occur. Arrhythmias are easy to detect with continuous ECG monitoring were atrial fibrillation (AF), tachycardia, and refractory ventricular fibrillation can be observed in FM. Therapeutic treatment options are recommended to follow current guidelines to treat arrhythmias. Implantation of temporary pacemakers or cardiac defibrillator (ICD) have demonstrated success and may be considered whenever necessary.^98^

### Pathophysiological processes in the long-term prognosis of FM

**Fig. 5:**
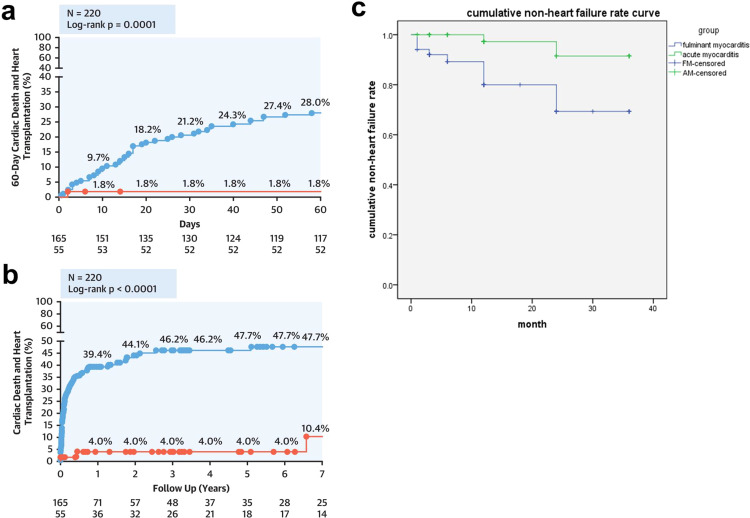
The prognosis of FM patients. **a** The 60-day follow-up and **b** long-term (7 years) follow-up of FM patients in the work of Ammirati.^45^ These two figures are of the permission of the *Journal of the American College of Cardiology*. **c** The cumulative non-heart failure rate curve of 105 patients who were treated at Tongji hospital between January 2015 and August 2019. These patients were diagnosed as acute myocarditis (*n* = 54) or fulminant myocarditis (*n* = 51). The primary endpoint was cardiac death or heart transplantation, and the secondary endpoint was left ventricular dysfunction evidenced by ejection fraction <55% during study follow-up. The Kaplan–Meier method was used to operate the survival curve. The heart failure rate of FM patients was significantly different from that of patients with acute myocarditis (Log rank = 0.009)

Currently, there are contradictory reports regarding the long-term prognosis of FM. It is commonly accepted the immune response to pathogens and the related disturbance in immune homeostasis has a marked impact on the long-term prognosis of FM. Although FM patients may be discharged with normal or slightly compromised heart function,^72^ disturbances of the immune system, remnant inflammation and changes in autoimmune tolerance influence prognosis and affect cardiac remodeling. We have found at 1-year follow-ups of FM patients, over 20% develop heart failure, arrhythmia or enlarged heart compared to ~10% of acute myocarditis (Fig. 5). Evidence suggests cytokines released from cytokine storms impact the cardiac remodeling process. For example, transforming growth factor beta (TGF-β), is a pro-fibrotic cytokine upregulated in the acute phase of FM, which will induce exorbitant fibrosis. Fibrosis and scar tissue also provide a structural basis for the occurrence of arrhythmia.^96^ A recent report described a 28-year-old male who rapidly progressed into dilated cardiomyopathy in 90 days after discharge from the hospital following FM and required a subsequent heart transplant. The explanted heart showed massive fibrosis.^99^ It is clear that the precise mechanisms of the long-term pathophysiological changes in FM remain unknown and, thus, need further investigation.

## Diagnosis and differential diagnosis of FM

The diagnosis of FM is largely based on the clinical symptoms observed in patients rather than a pathological or pathophysiological diagnosis.^2^ The following criteria should be met to diagnose FM: (1) rapid onset of symptoms of severe heart failure (quick deterioration of EF% or newly occurrence of transduction block) within 2 weeks; (2) prodromal symptoms of upper respiratory or gastrointestinal viral infections; (3) rapid development of hemodynamic compromise requiring large doses of inotropic drugs, like dopamine, dobutamine, and even MCS devices; (4) CMR- or EMB-proven myocarditis (discussed below); and, (5) exclusion of other cardiac diseases, especially acute ischemia cardiomyopathy or coronary artery disease.^1,4,45,100,101^ Importantly, it is critical to differentiate between FM and acute coronary artery disease as the treatment regimens are completely different. Once a previous healthy individual or a non-cardiac disease patient shows rapid heart failure, it is very important to consider the possibility of FM.

EMB is considered the golden standard for diagnosis and helps the pathological classification of FM. While not every patient undergoes EMB, recent data indicate the rate of EMB use to diagnose FM has increased.^101^ In Europe, about 20–50% of FM patients undergo EMB,^41^ whereas a registry of FM patients in the USA demonstrated an EMB rate of <5%.^102^ Although performing EMB requires tertiary clinical centers with specialized equipment and personnel limiting patient assessment, it is strongly recommended to perform EMB as soon as possible. With the application of modern molecular biological techniques, such as RT-PCR, ISH or NGS,^18^ EMB samples can provide key information about FM. The safety of EMB can be guaranteed by an experienced operator and the incidence of adverse effects were reported to be <2.5%.^1,42^ The majority of complications are not lethal, including tricuspid regurgitation, transient right bundle branch block, and transient arrhythmia. Life-threatening complications include ventricular perforation and related cardiac tamponade.^41^ Another concern about EMB is that the inflammatory infiltration of the heart may not be uniform, so EMB might give false-negative results. It has been reported that by getting 4–5 or even more EMB samples, the sampling error and false-negative results could be reduced.

CMR is an alternative choice if EMB is not accessible or the situation of the suspected FM patient is not stable. CMR can provide physicians with data about both heart function and cardiac morphology. The ability to clearly distinguish the ventricular wall from the blood chamber can provide a better resolution of morphology and precise evaluation of heart function. With the application of different scanning sequences with or without contrast enhancement, CMR is able to show good tissue characterization and visualize pathological tissue changes, including intracellular and interstitial edema, hyperemia, capillary leakage and even necrosis and subsequent tissue fibrosis (Fig. 4).^103^ The most commonly used contrast enhancement agents are gadolinium-derived. Early gadolinium enhancement is a sign of hyperemia and capillary leak, while late gadolinium enhancement (LGE) is a sign of necrosis and fibrosis.^73^ When using CMR to diagnose myocarditis, Lake Louise Criteria are the most widely accepted criteria to follow for the final diagnosis.^73^ According to a recent study, the LGE pattern of FM was significantly different from that of non-fulminant myocarditis (NFM) (Fig. 4), which could help make a differential diagnosis.^104^

Although CMR is capable of providing physicians with detailed information about pathological morphological changes and heart function, its most important shortcoming is the requirement of special equipment and a time-consuming scanning process, which limits its application in emergency and clinical centers that are not capable of performing CMR because of emergency conditions of patients and its requirement for heart rate control. In contrast, emergency echocardiography is able to provide information about heart function and cardiac contraction status (globally hypokinesis) quickly and is suggested as a first-line assessment. We have found in FM that strains among different layers of the myocardium were diffusedly impaired,^72^ while NFM data mimic AMI and display segmental impairment of myocardium strain.^105^ This feature of FM may also provide information to differentiate FM from NFM.

Signs and symptoms like fever, palpitation, sore throat, syncope and arrhythmia could be common but unspecific prodromal signs of FM.^106^ Because of these unspecific prodromal manifestations of FM, it is important to make a careful differential diagnosis in order to avoid inappropriate treatments. Apart from acute myocardial infarction, FM should also be differentially diagnosed from Tako-Tsubo cardiomyopathy, pneumothorax and even acalculous cholecystitis.^107^

## Treatment regimen of FM

FM presents as a rapidly progressing severe condition where patients respond poorly to conventional vasoactive drug therapies as well to standard heart failure, refractory heart failure, and cardiogenic shock treatments. However, improvements in MCS devices has resulted in better therapeutic success in treating FM patients from being <20% to 40–70%.^6,108–110^ Recently, FM patients in our center were successfully treated with the “life support-based comprehensive treatment regimen”,^4^ which significantly reduced FM mortality from ~50% to <5% and shortened the hospitalization period to <2 weeks. Similar beneficial effects have been demonstrated in other centers in China^8^ using this therapeutic approach.

How did the treatment regimen evolve from being a simple drug therapy to comprehensive treatment regimen? What are the differences among these regimens? It there any possibility of further improvement?

### 1980s–1990s: the age of drug therapy for FM

Due to limited circulation support and monitoring devices, FM was very hard to distinguish from common myocarditis during this period.^111^ The only remarkable symptoms were a quick deterioration of heart function and severe heart failure. Hence, it was very reasonable to use standard heart failure therapy to treat FM owing to limited interference strategies. Positive inotropic drugs or vasoactive drugs were the first line of drugs administered to enhance heart pump function and act against the decreased blood pressure. However, high in-hospital mortality indeed announces a failure in simple drug therapy.^112^

Results from pathological findings identified massive immune cells, like lymphocytes, infiltrating myocardium, leading to the utilization of immunosuppressive agents such as cyclosporine, azathioprine and tacrolimus.^113–115^ Additional approaches included administration of glucocorticoids and IVIG to suppress the immune response.^112^ However, the effects of these immunosuppressive agents remain debatable. For example, results from ‘The Myocarditis Treatment Trial’ failed to support immunosuppressive therapy as a routine treatment for myocarditis and suggested long-term mortality of immunosuppressive therapy was high.^115^ In this clinical trial, the mean change in LVEF were not significantly different between the immunosuppressive group and control group (+0.10 vs. +0.07, *p* > 0.05), and the mortality rates of the two group were also not significantly different throughout the follow-up (65% of control group vs. 55% of the immunosuppressive group, *p* = 0.96). Numerous limitations of the study included reliance on the ‘Dallas Criteria’ to distinguish biopsy samples with an EMB rate of only 10%, an inadequate sampling amount, enrollment of patients with EF% <45%, which indicated poor recovery from previous treatment, and insufficient dosage of immunosuppressive agents.^116^ Taken together, standard heart failure treatment aiming to improve cardiac pump function or immunosuppressive treatment, targeting infiltrating immune cells, showed little effects in FM, which set barriers for patients.

### 1990s–2010s: the age of MCS therapy in FM

The development of extracorporeal circulation devices to allow the pump function of the heart to be temporarily replaced by a mechanical device contributed to rapid advancements in cardiac surgery. These new technologies led to further development of different, compatible, and convenient MCS devices that could be rapidly applied to cardiology treatments, including FM, markedly improving outcomes.

The predominant physiological change observed in FM patients is hemodynamic instability, which causes a sharp decrease in blood pressure, low infusion of important organs and finally shock. Although there are several different kinds of MCS devices currently in use, the principle of MCS is to provide the patient with the indispensable circulation support to avoid shock.

Intra-aortic balloon pump (IABP) is the most commercially available MCS device being used in treatment, where a balloon synchronously inflates and deflates with a systolic and diastolic heart rhythm.^117^ Thus, IABP can lower LV afterload and increase the blood flow to the brain and kidneys. However, due to the limited size of the balloon and less power of the pump, IABP can only provide about 15% of extra circulation support compared with the total circulation demand. In our practice, it is good enough for most FM patients and if not enough, additional mechanical support tool, such as extracorporeal membrane oxygenation (ECMO) should be added.

ECMO is another useful MCS device to provide more powerful circulation support. The flow of ECMO can be adjusted from 0.5 L/min to 4.5 L/min, which can meet the basic demand of the body circulation. ECMO has two different working modes. The venous to arterial (VA) mode is designed to support body circulation and organ infusion due to refractory heart failure.^101^ While the venous to venous (VV) mode is used to support pulmonary circulation and provide oxygenation to venous blood when acute respiratory dysfunction syndrome (ARDS) presents.^118^ VA-ECMO can effectively lower the preload of the heart by 40–60%^119^ and, therefore, is increasingly being utilized for treating FM. In our clinical observation in more than 100 FM patients, about 75% received only IABP and for the remaining patients ECMO was added over IABP, and thus the circulation was maintained well.^8^

Several other devices have been developed such as Impella, which is a small pump sent into the LV to drain blood and decrease load.^88,120,121^ However, limitations in an ability to reach high flow rates and limited commercial availability restrict the wider application of Impella. In a recent study, comparing the in-hospital mortality rate and major bleeding between Impella and IABP among acute myocardial infarction patients with cardiogenic shock, use of Impella was associated with a higher in-hospital mortality and major bleeding rates.^122,123^ In contrast, evidence also suggested a trend of improved outcomes in Impella supported patients.^124^ Together, this suggests a need for caution when considering the usage of Impella for FM patients. Other approaches employed to treat FM involve Ventricular Assistance Devices (VAD), including Left-VAD (LVAD), Right-VAD (RVAD), and Bi-VAD.^125–127^ In addition, an artificial heart has also been reported to be applied to assist circulation and bridge FM patients to heart transplantation.

While MCS devices have provided much-improved assistance in the treatment of FM and help lower the mortality rate, there remain limitations as they do not entirely provide the circulation support of natural hemodynamics. For example, the VA mode of an ECMO pump provides oxygenated blood directly into the descending aorta from the femoral artery in the opposite direction of the natural blood flow potentially causing endothelium injury due to turbulence and increased LV afterload. Long-term usage may lead to thrombosis, abnormalities of coagulation and infection of ECMO loops. Impella mimics the natural blood flow direction but due to its limited flow capability and high cost, there is limited usage outside Europe. Due to the underlying risk of life-threatening thrombosis or hemolysis, MCS devices are usually applied as the bridge to recovery or further heart transplantation.^128^

Advancements in FM treatments following the development of MCS devices have resulted in increased survival rates.^6^ However, it is important to recognize the benefit of successful co-therapies using proper drugs with MCS devices. Indeed, a majority of clinical cases where MCS were applied successfully to FM patients also showed usage of inotropic drugs like dopamine and dobutamine. It should be noted the success rate of MCS could only reach 40–70%, in a few reports it could reach higher than 80%.^6,7,108^ Considering the residual mortality, we further speculate about the other processes in FM except for “pump failure”.

### 2010s–present: the age of comprehensive treatments for FM

Our increased knowledge about the underlying pathophysiology of FM, revealed it is a severe disease characterized by acute heart failure and the occurrence of a serious inflammatory response. An investigation into the etiology of FM demonstrates disturbances in the immune system, overstimulation, and disrupted cytokines and chemokines profiles, is critical.^129^ Upregulated cytokines or chemokines are the natural reaction against infection but a huge disturbance of immune homeostasis can lead to organ damage. Pro-inflammatory cytokines like IL-1, IL-6, CD-40, and TNF-α, as well as and anti- inflammatory cytokines including sTNFR and IL-10, are elevated in the serum of FM patients.^130^ These changes observed in immune responses have been be replicated in FM animal models.^131,132^

Disruption of the immune response in FM, especially T-lymphocyte infiltration, can be observed in the majority of EMB or postmortem samples. Moreover, the development of FM is promoted by the over-activated immune response against those triggers. Massive amounts of cytokines, both pro- and anti-inflammatory, are released by locally infiltrated immune cells and from other organs, which further destroy the immune environment of the myocardium.^56,131^ We have determined the plasma cytokine profiles of FM patients and found that over 30 cytokines were upregulated and over 10 cytokines were downregulated, referred to as a “cytokine storm” (Fig. 2). Interestingly, sST2, the decoy receptor of IL-33, was significantly increased when FM patients were admitted and gradually decreased to normal levels the patients improved. Consistent with human data, animal experiments demonstrated normal mice treated with sST2 had decreased heart function, while experimental FM mice showed better survival rates when treated with an anti-sST2 antibody (100% of anti-sST2 antibody VS 50% of FM mice). In line with our data, it was reported plasma pro-inflammatory cytokine levels were downregulated when MCS devices were applied to the patients and hemodynamic conditions improved,^121^ which indicated the hemodynamic state is tightly associated with the cytokine levels. However, the underlying mechanisms need further investigation.

**Fig. 6:**
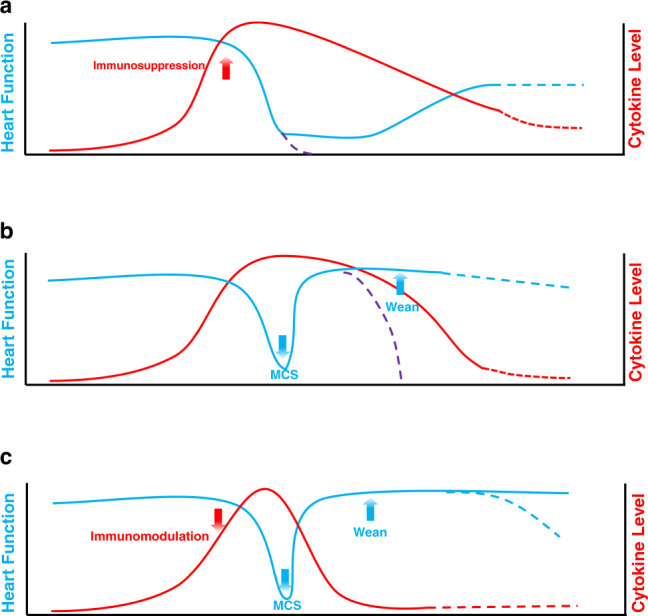
Illustration of different regimens to FM. The age of drug therapy (**a**), the age of MCS therapy (**b**), and the age of comprehensive therapy (**c**) carry out different interference methods. The red line represents cytokine level while the red dot line represents possible cytokine level. The blue line represents heart function, and the blue dot line represents possible future heart function manifestations. Note that heart function may deteriorate and lead to death (purple dot line)

By considering the current understanding of FM, especially the roles of the cytokine storm, we established a new regimen termed “life support-based comprehensive treatment regimen” to treat FM.^4^ We emphasize the combined use of MCS and other life support devices, including mechanical ventilator and hemodialyzer, together with approaches to balance the disturbed immune response by immunomodulation therapy using sufficient dosage of glucocorticoids (usually 200–400 mg or higher dose of methylperidenolsone per day for few days) and IVIG but not cytotoxic agents, such as cyclosporine or azathioprine (Fig. 6). During the period of drug therapy (Fig. 6a), the application of cytotoxic drugs could only gradually downregulate the cytokine levels. However, without the administration of immunomodulation therapy by glucocorticoid and IVIG, the protective such as the promotion of nitric oxide production, attenuation of myocardium edema, and elevation of cardiomyocyte survival effects by glucocorticoid and beneficial effects from IVIG were absent.^133^ As a result, the application of cytotoxic drugs could not promote the survival of FM patients, as The Myocarditis Treatment Trial proved. Due to insufficient circulation support, heart function may not able to recover rapidly and can be overwhelmed by the cytokine storm leading to death. The application of MCS will immediately improve heart function, however the lack of control of cytokines costs more time to wean out MCS devices or eventually lead to death (Fig. 6b). The combination of immunomodulation and MCS, which is the core of the “life support-based comprehensive treatment regimen”, supports dysfunctional circulation and attenuates an overwhelming cytokine storm (Fig. 6c).

**Fig. 7:**
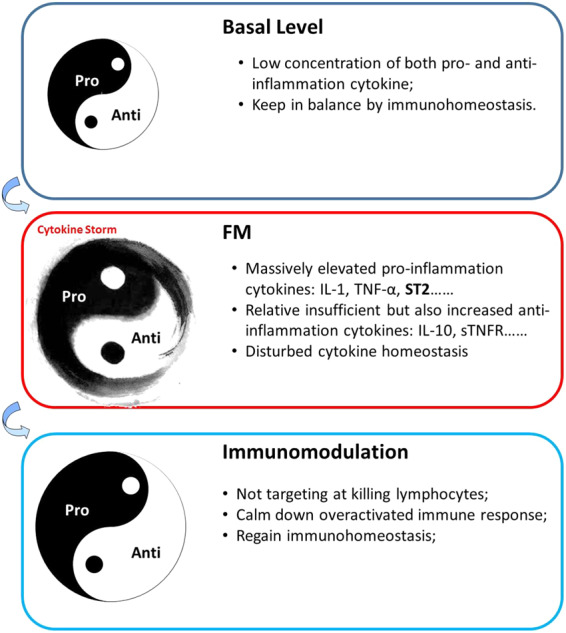
Illustration of the relationship of cytokine storm and immunomodulation. The cytokine storm does not necessarily equal to the elevation of absolute quantification of certain cytokine, but is more likely to be the disturbance of immunohomeostasis. The effect of immunomodulation is to restore immunohomeostasis and calm down overreacted immune response

It should be noted that in the “life support-based comprehensive treatment regimen”, sufficient doses of both glucocorticoids and IVIG were defined as immunomodulation agents, but not immunosuppressive agents. Meanwhile, pure immunosuppressive agents or cytotoxic, such as cyclosporine and azathioprine, which mainly target lymphocytes were not recommended. Numerous reports revealed the effectiveness of using IVIG in treating FM patients by modulating the immune response and neutralizing pro-inflammatory cytokines.^132,134^ In our experimental FM animal models, application of cyclosporine showed no benefit to the survival of myocarditis in mice (40% of cyclosporine VS 30% of myocarditis) but IVIG prevented death when administrated either before or after the onset in C57 mice (model for myocarditis) or A/J mice (model for FM). The application of glucocorticoids remains debatable and the best timing for administration of glucocorticoids remains unknown. It was reported in an animal model of acute viral myocarditis the different timepoints of glucocorticoid administration results in the different mortality rate of mice.^135^ It should be noted that although the early application of glucocorticoid was mainly aimed at controlling the cytokine storm rather than suppressing the overall immune response, there is a concern it will lead to enhanced viral spread. Previous recommendations suggest immunosuppressive therapy be considered only whenever the virus load is negative in myocarditis.^136^ Evidence indicates the administration of glucocorticoids can significantly improve the prognosis of patients with EMB proven lymphocytic myocarditis, in either virus-positive or virus-negative individuals.^137^ Hence, there remains a debate about the effectiveness of glucocorticoids in FM. It is still recommended that the application of corticoid must be with caution at the stage of viremia. However, at the critical stage with critical illness, the major pathophysiology is the cytokine storm rather than the viremia, and according to our data, the majority of patients had already experienced the onset of a cytokine storm on admission and urgently required immunomodulation therapy. Too much caution regarding the possibility of enhancing virus spreading by glucocorticoid may delay live-saving treatment. Experimental studies suggest the application of glucocorticoid to A/J mice infected with CVB3 lowered the viral titer in heart tissue.^8^ Clinical evidence also indicated that the application of glucocorticoid in virus pneumonia and recent COVID-19 is safe and effective.^138^ Therefore, it can be proposed application of immunomodulatory therapies aim to restore the homeostasis of the immune system (Fig. 7).

Another important aspect of the “life support-based comprehensive treatment regimen” is anti-virus therapy. Although various etiologies may result in FM, viral infection is still considered as the predominant trigger. During the H1N1 flu pandemic in 2009, several case reports indicated anti-viral agents such as oseltamivir and zanamivir had encouraging therapeutic effects.^139–141^ The beneficial effect may be related to the neuraminidase inhibitor properties, where neuraminidase released from the injured heart is thought to be detrimental to the heart itself.^142^ If the certain infected virus can be confirmed, targeted anti-viral drugs can be applied. Other beneficial agents have been indicated to have dramatic effects against viral myocarditis. For example, free immunoglobulin light chain showed antiviral and antiinflammation effects in an animal model of viral myocarditis, which provided support to the clinical application of IVIG.^143^ Therapeutic agents that can actively neutralize viral particles and demonstrate potential anti-viral properties towards targets such as soluble coxsackie- and adenovirus receptors^144^ provide insight into novel therapies. Thus, considering the role of viral infection in FM, it suggested anti-viral therapies be considered when making clinical treatment decisions.

**Fig. 8:**
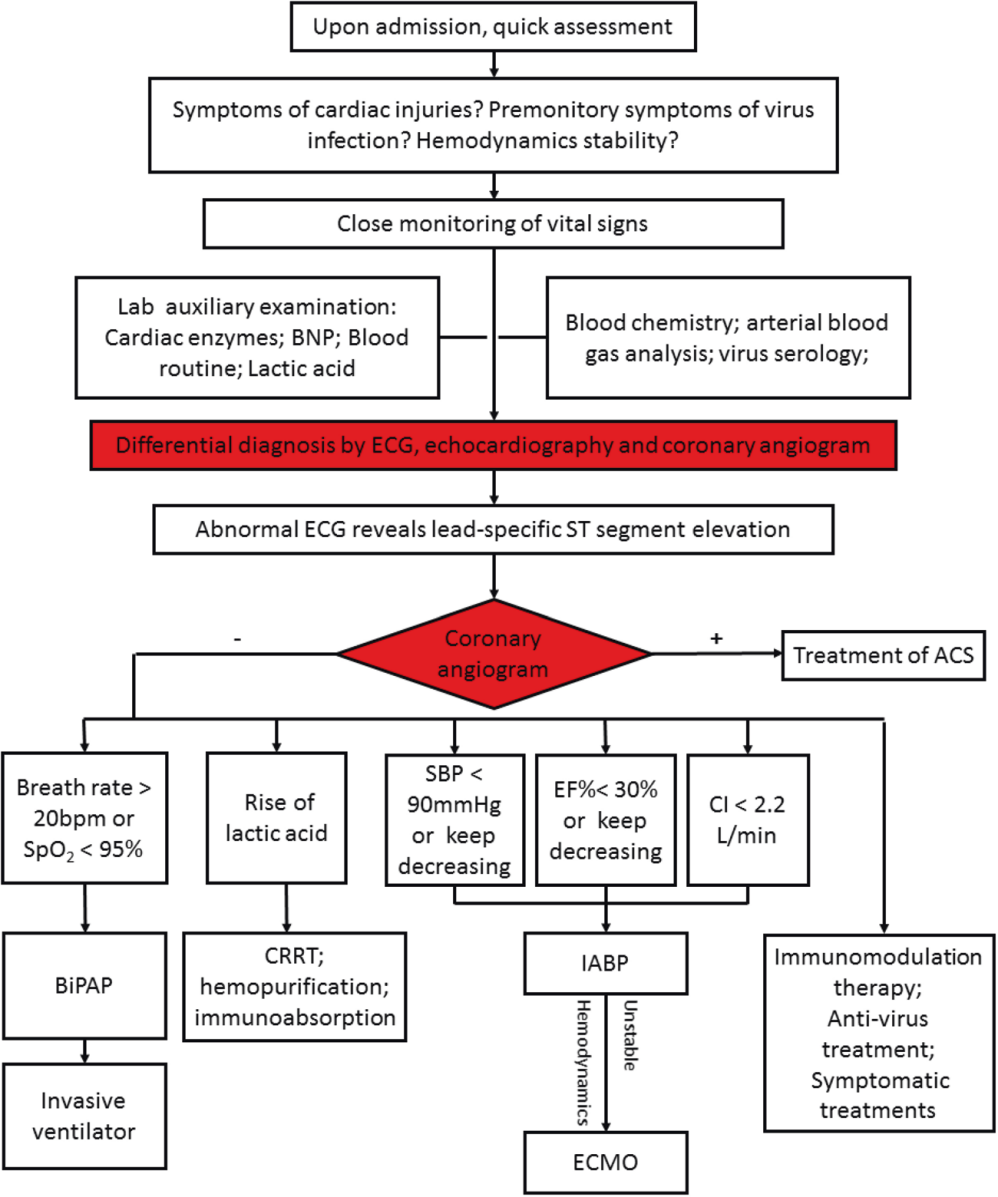
Flow chart of treating FM under the guidance of “life support-based comprehensive treatment regimen”

As mentioned above, the core concept of the “life support-based comprehensive treatment regimen” is to modulate the immune response and provide circulation support to the deteriorated hemodynamic state via MCS. We prefer applying IABP as the first-line MCS device. While several reports reported the benefit of Impella in treating cardiogenic shock, recent studies revealed that Impella might be associated with a higher risk of major bleeding and in-hospital mortality, accompanied by a decreased cost-effectiveness value.^122,123^ There are several other treatments including anti-viral and anti-arrhythmia therapies or using a neuraminidase inhibitor, like oseltamivir, to prevent damage to the myocardium by the released neuraminidase. A recent study indicated that neuraminidase is a potential cardiac detrimental factor and a related clinical trial is being carried out to determine the effectiveness of oseltamivir in FM (NCT03268642, https://clinicaltrials.gov). The flow chart illustrates the process followed when treating FM patients according to the “life support-based comprehensive treatment regimen” (Fig. 8). It is important to note adaptations to treatments must be made in accordance to the current and changing status in an individual patient’s health.

## Prognosis of FM

There are contradictory reports in the literature regarding the prognosis of FM. For example, in 2000, McCarthy et al. published data from a single-center research study, which indicated FM had a better prognosis than acute (non-fulminant) myocarditis.^102^ Conversely, Ammirati et al. recently published a study demonstrating FM, confirmed by EMB and classified into three subtypes, exhibited worse outcomes than non-FM in 60-day outcomes and long-term follow-up,^45,145^ as the 60-day mortality rate of FM patients is 28% compared with 1.8% of non-FM patients (*p* = 0.0001), and the long-term follow-up (7 years) showed 47.7% of FM patient mortality rate compared with 10.4% of non-FM patients, respectively, (*p* < 0.0001) (Fig. 5).

Numerous differences in how each study was designed to provide some insight into the opposing results in these reports. In the first study, McCarthy’s group enrolled patients from 1984 to 1997 and among the 147 enrolled patients only 15 met the criteria of FM; while Ammirati enrolled 165 EMB-proved FM patients and 55 non-FM patients from 2001 to 2018 resulting in a more powered study. The EMB percentage of FM in McCarthy’s study remained below 5% along the whole enrollment for the study and non-FM decreased from about 20% to <5%. Importantly, the time period between administration and EMB was shorter in Ammirati’s study than McCarthy’s, which means persistent lymphocyte infiltrate after the acute phase of FM. And MCS devices in McCarthy’s study were LVAD, without IABP or ECMO, which might be due to the conditions at that time. In the recent study, IABP, ECMO, and Impella were all considered when stable hemodynamics cannot be sustained.^8^ Together, the low EMB rate and restricted MCS device choice contributed to differential and misleading pathological diagnosis and potentially a wrong prognosis.

In contrast, in our multi-center observational study, we compared the in-hospital mortality of 169 FM patients. Of them, 81 patients were treated with the “life support-based comprehensive treatment regimen” while the other 88 patients were treated with the traditional therapy defined as “carrying out stepped regimen of conventional medicines for heart failure and cardiogenic shock”, e.g. vasopressor and positive inotropic drugs, no MCS devices, no immunomodulation therapy or delayed the application of MCS until the circulation of FM patients collapsed; MCS devices, e.g. IABP, were only successively applied if pharmacological therapy could not maintain circulation stability.^8^ Results demonstrated the “life support-based comprehensive treatment regimen” could significantly lower the mortality rate from 46.6 to 3.7% of all enrolled patients, and from 42.1 to 2.6% of propensity-matched patients. These data provide evidence supporting the comprehensive approach in the treatment of FM and reflect experiences from multiple clinical centers in China.^8^ It has been considered “of great value and can help clinical workers treat and save more lives successfully from FM”.^146^

Our therapeutic approach is supported by Ammirati’s work by placing an emphasis on the importance of both MCS and immunomodulation therapy.^45,147^ We propose this is the key to a successful treatment of FM. However, there are some differences between the two regimens. In our regimen, we do not recommend the use of inotropic drugs like dopamine or dobutamine, at least, not as dominant treatment, but MCS devices to maintain circulation and suggest a different application rate of glucocorticoids (100% vs. 24%), IVIG (100% vs. 34%) and neuraminidase inhibitor (100% vs. 0%). In addition, we did not recommend use of cytotoxic drugs like cyclosporine and azathioprine to suppress the immune system. Moreover, our 1-year follow-up results (Table 2) suggest no FM patient death was observed, supporting the idea that treatment by “life support-based comprehensive treatment regimen” improved the long-term outcome of FM patients. Among them, 41 of 51 patients (80.4%) showed fully recovered heart function, but there were still 10 of 51 patients (19.6%) showed decreased EF and enlarged left ventricular chamber or arrhythmias, which indicated heart failure. However, the main limitation of our work is the lack of EMB results, which hindered the pathological classification of FM.

**Table 2:** Clinical outcomes of fulminant myocarditis

Institution, Year	Follow-up	Myocarditis type	Outcome
Johns Hopkins Hospital et al., 2000^102^	5.3 years for FM 5.6 year for acute myocarditis	Pathologic diagnosed lymphocyte FM = 15 NFM = 132	FM: 1 death/0 HTx NMF: 59 death/11 HTx
Johns Hopkins Hospital et al., 2000^154^	6 months	Pathologic diagnosed myocarditis FM = 11 Acute Myocarditis (AM) = 43	FM: improved FS% Acute Myocarditis: No improvement
Niguarda Hospital et al., 2019^45^	60 days 7 years.	Pathologic diagnosed Lymphocytic: FM = 120; NFM = 39 Giant cell: FM = 19; NFM = 2 Eosinophilic: FM = 24; NFM = 10	60 days: FM: 39 death/7 HTx NFM: 1 death/0 HTx 7 years: FM: 47 death/24 HTx NFM: 4 death/1 HTx
Puerta del Mar University Hospital, 2019^155^	1 year	Clinical diagnosed FM = 12 NFM = 30	Poor outcome (Death, transplant or LV systolic dysfunction/dilation after 1 year of follow-up): FM: 5/12 (41%) NFM: 2/30 (7%)
Tongji Hospital, 2020. (This Review)	in-hospital 1 year	Clinical diagnosed FM = 51 AM = 54	in-hospital: FM: 0 death/ 0 HTx AM: 0 death/ 0 HTx 1 year: FM: 0 death/0 HTx AM: 0 death/0 HTx, but FM shows higher heart failure rate

## Future perspectives regarding the investigation of FM

It should be emphasized FM remains primarily a clinical diagnosis or clinical syndrome making it difficult to establish appropriate experimental animal models. Presently, the most widely used FM model is virally induced (usually CVB3) FM in A/J mice. However, a viral infection, although it is the most frequent etiology, is not the only etiology of FM restricting the translational interpretation. Moreover, multiple different types of viruses, besides CVB3, can induce FM. In addition, it is technically difficult and expensive to routinely monitor hemodynamics and utilize MCS in mice.

Although the fundamental pathophysiological mechanisms underlying FM are far from thoroughly revealed, the disturbance of the immune system plays an important role in the development and progress of FM. The evolution of treatment regimens provide evidence emphasizing the importance of addressing the immune response in treating FM. Unfortunately, there is a lack of experimental models and evidence for determining whether the pathogens directly initiate the cardiac immune response or if the injured myocardium and subsequently released contents induce the immune response. The involvement of a cytokine storm has been confirmed in various clinical studies and animal models. The disrupted cytokine response is potentially an important therapeutic target. The established treatment regimens still rely on global immunomodulation or immunosuppression, like that induced by IVIG or steroid use, instead of relying on precise target interference. Recently, evidence for therapy targeting precise cytokines has been reported^148,149^ and have been excellently reviewed elsewhere.^150^ Although there are different pathological classifications of FM, whether there is a common immune response or key cytokine(s) is still worthy of investigation. Furthermore, target therapy to special cytokine(s) may be promising approach.

The long-term outcome of FM has an important impact and requires special attention. We and other researchers have observed cardiac outcomes in a considerable proportion of surviving FM patients often progress to dilated cardiomyopathy (DCM) with decreased EF.^99,113^ It is believed the chronic immune response has a major role in the adverse progression of FM. The presence of anti-cardiac antibodies such as anti-MHC can be detected in the serum of these patients, and is associated with decreased left ventricular ejection fraction (LVEF).^151^ The strategy to prevent this process and which kind of immune cells take part in this process need to be determined.

## Conclusion: the beginning of a new era and exploration

If we look back to the history of FM, we can find treatment regimens have evolved from drug therapy, only targeting the pump function of the heart, to a current “life support-based comprehensive treatment” regimen, which emphasizes the combination of MCS devices with modulation of the immune system. The success of the approach has led to a reduction in the mortality to <5%.^8^ This breakthrough in therapy for FM treatment is easily adapted in the clinic and *“*has the potential to save the lives of many young, otherwise healthy individuals”.^152^

However, the mechanisms underlying FM are still not fully revealed and the best treatment regimen for recovering patients remains to be elucidated. During the acute phase of FM, the future investigation should be focused on the combined management of circulation stability and immunomodulation, which can sustain the basal circulation of the FM patient as well as lower the immune disturbance. New MCS devices capable of providing more natural hemodynamic circulation support may be needed or the ones available may need to be upgraded. Clinical trials should be performed to develop agents or antibodies targeting the elevated cytokines and achieving a precise interference of elevated cytokines. For discharged patients, the long-term cardiac remodeling process is a key issue to be focused on. The true long-term prognosis of FM patients still needs to be determined in future follow-up studies, and the treatment regimen for these patients should be determined by clinical trials.
